# Supplementary material for: Vagal determinants of exercise capacity
Source: Nat Commun. 2017 May 18;8:15097. doi: 10.1038/ncomms15097 (PMC5454375; doi:10.1038/ncomms15097)
Supplement: Supplementary Information — Supplementary Figures and Supplementary Tables. [file ncomms15097-s1.pdf]

Supplementary Information

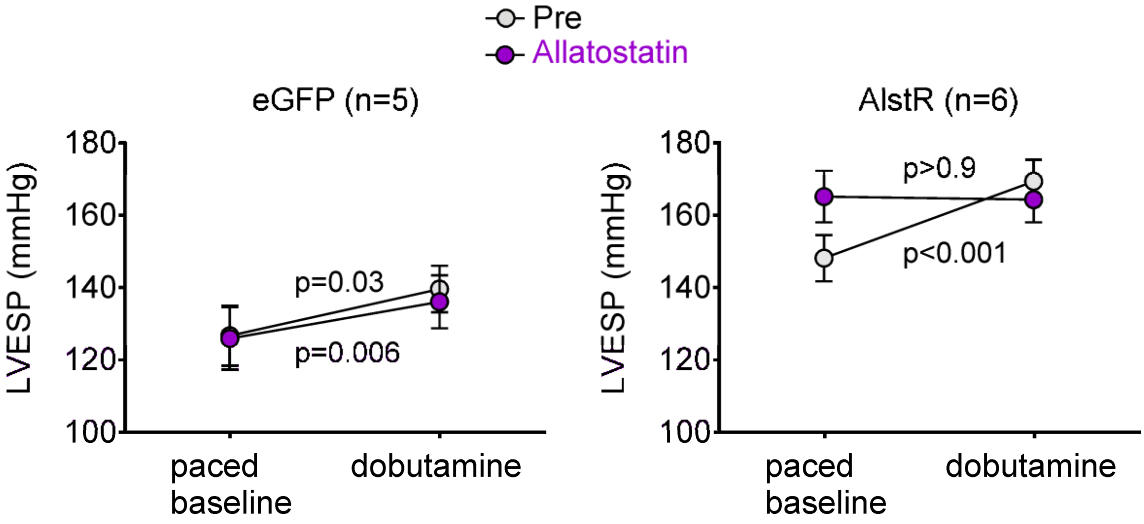

Supplementary Figure 1| Summary data illustrating changes in left ventricular end-systolic pressure (LVESP) in response to β-adrenoceptor stimulation (administration of dobutamine) at baseline (paced at 20% above the resting heart rate) and 15 min after allatostatin infusion into the *cisterna magna* in rats transduced to express enhanced green fluorescent protein (eGFP, n=5) or allatostatin receptor (AlstR)/eGFP (n=6) by the DVMN neurons. Data are presented as means±s.e.m. Comparisons are made using ANOVA.

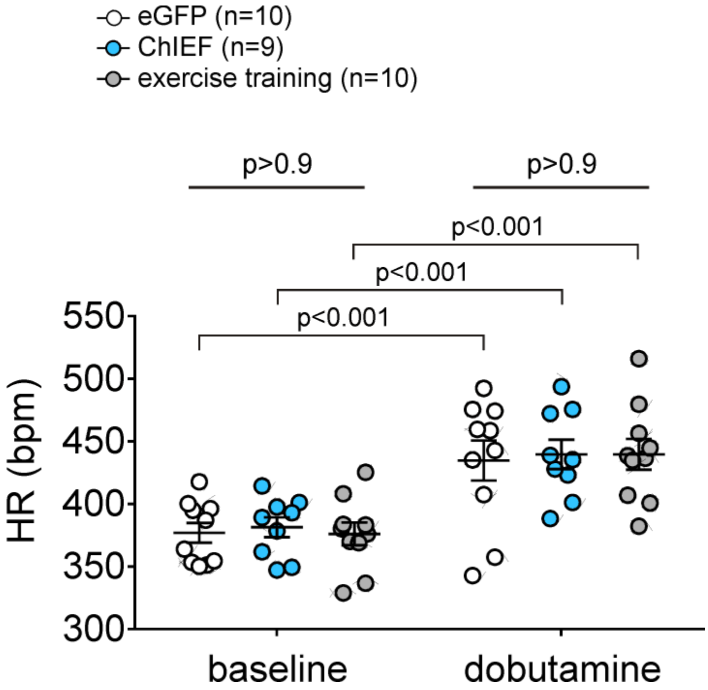

Supplementary Figure 2| Summary data illustrating baseline heart rate and chronotropic responses to β-adrenoceptor stimulation (administration of dobutamine) in rats transduced to express eGFP (n=10) or ChiEFtdTomato (n=9) by the DVMN neurons after 4 days of 445 nm light stimulation of the dorsal brainstem and in a group of naïve animals (n=10) following 4 daily sessions of treadmill exercise training. Data are presented as individual values and means±s.e.m. Comparisons are made using ANOVA.

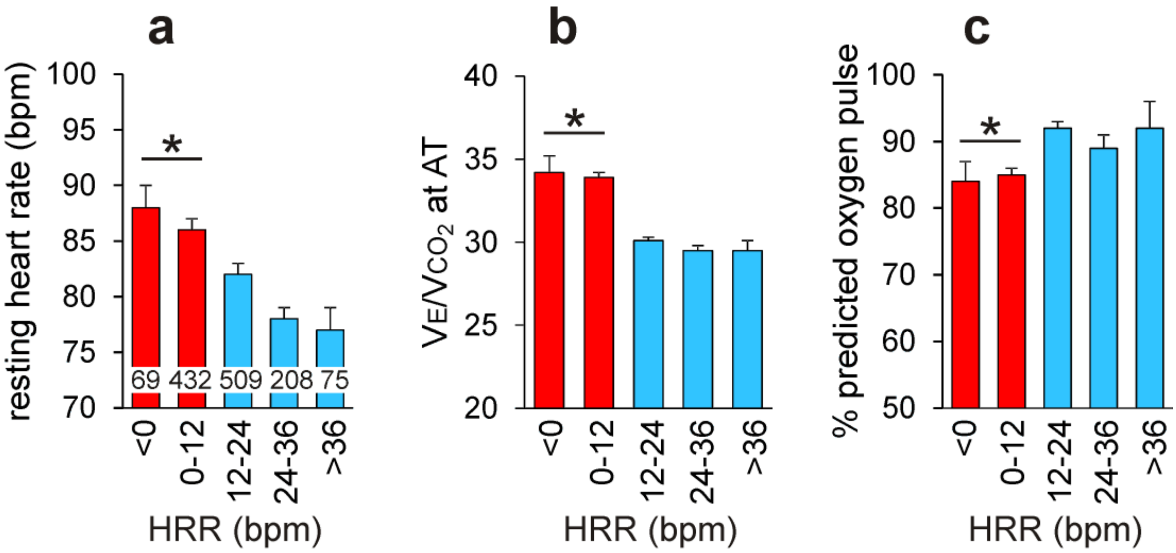

Supplementary Figure 3| Exercise capacity is reduced in subjects with cardiac vagal dysfunction. (**a**) Resting heart rate in subjects (n=1293), stratified by heart rate recovery (HRR) 1 minute after cessation of cardiopulmonary exercise testing. Numbers of subjects represented within bars. Resting heart rate is higher in the participants with reduced HRR following exercise. (**b**) Minute ventilation-carbon dioxide production relationship (V_E/VCO_2 slope) is higher in subjects with lower HRR. (**c**) Percentage of predicted oxygen pulse achieved is reduced in conditions of vagal dysfunction. Data are presented as means±s.e.m. *p<0.001 (ANCOVA/ANOVA, with post-hoc Tukey-Kramer testing).

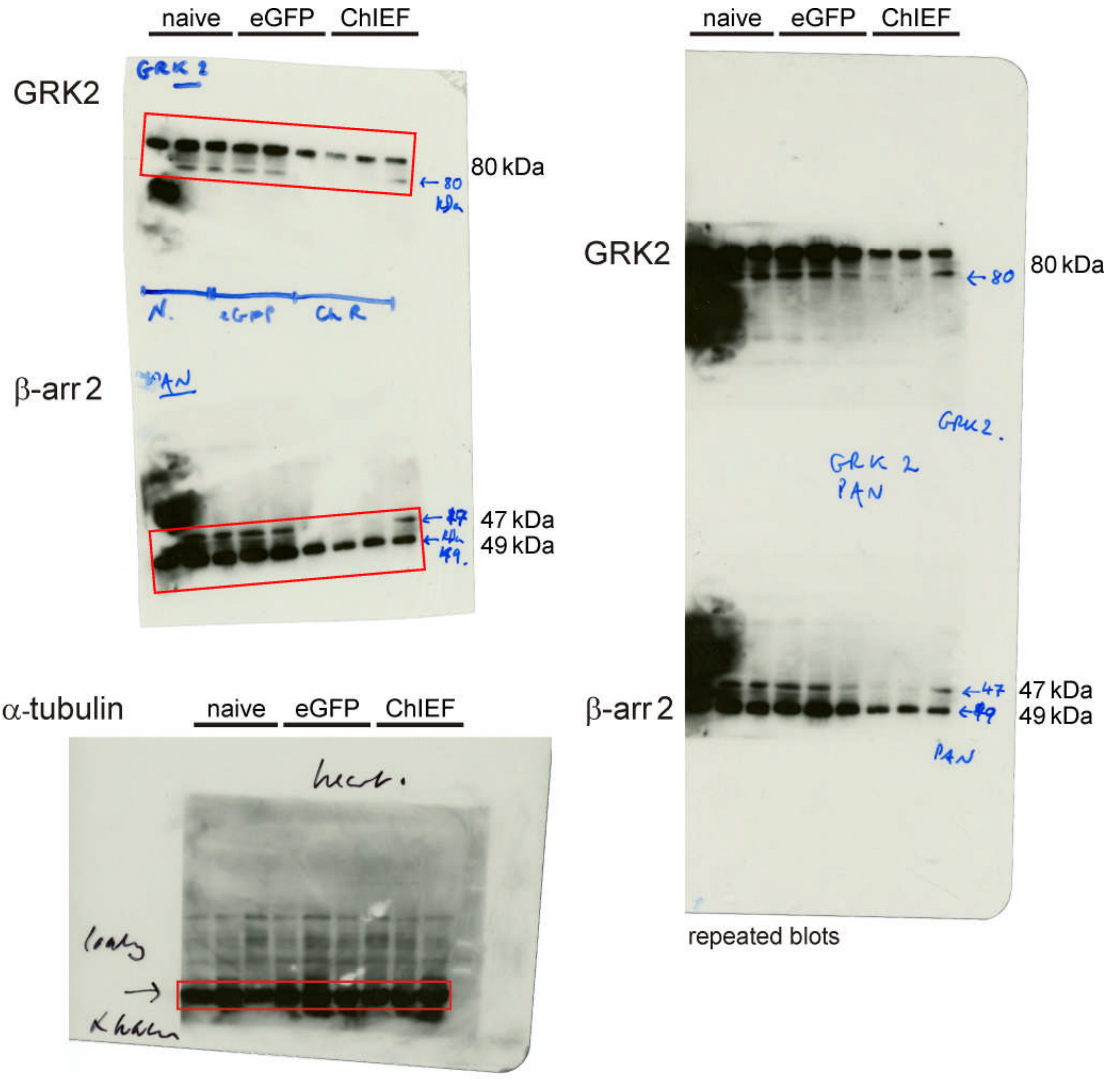

Supplementary Figure 4| Uncropped images of the western blots.

Supplementary Table 1

Characteristics of population undergoing cardiopulmonary exercise testing (1293 human participants)

Age: 63±14

Gender: 67% male

Body-mass index: 26.9±5.3

Cardiovascular disease: 45%

Diabetes mellitus: 11.7%

Resting heart rate: 83±16 beats.min^{-1}

Peak heart rate: 135±24 beats.min^{-1}

% predicted peak VO_2: 74.7±23.8%

% predicted oxygen pulse: 88.7±28.0%

Ventilatory equivalents, CO_2 at anerobic threshold: 31.5±5.8

Anerobic threshold: 11.3±3.2 ml.kg^{-1}

ST segment depression during exercise, lead II: -0.6 (-0.3 to -1.1) mm

Borg exertion scale: 15 (14-17)

Duke activity score: 43 (27-54)

Supplementary Table 2

Multivariate logistic regression analysis of factors associated with delayed HRR. On multivariate logistic regression analysis, the only factors associated with delayed HRR were age and diabetes mellitus. Multivariable logistic regression analysis showed no association with any cardiovascular medication.

Independent variable	Regression Coefficient	Standard Error	Wald Z-Value	Wald P-Value	Odds ratio
Intercept	-2.14	0.55	-3.88	0.00	0.12 (0.04-0.35)
Age	0.02	0.01	3.71	<0.001	1.02 (1.01-1.04)
Body-mass index	0.02	0.02	1.00	0.32	1.02 (0.99-1.05)
Female gender	0.23	0.17	1.37	0.17	1.26 (0.90-1.76)
Beta-blocker	0.26	0.25	1.04	0.30	1.30 (0.79-2.12)
ACE inhibitor/ARB	0.12	0.23	0.50	0.62	1.12 (0.71-1.77)
Statins	0.00	0.21	0.01	0.99	1.00 (0.67-1.51)
Diabetes mellitus	0.60	0.25	2.43	0.01	1.82 (1.12-2.94)
Cardiovascular disease	0.13	0.22	0.60	0.55	1.14 (0.74-1.76)
